# The Ability of Hop Extracts to Reduce the Methane Production of *Methanobrevibacter ruminantium*

**DOI:** 10.1155/2021/5510063

**Published:** 2021-11-05

**Authors:** J. A. Blaxland, A. J. Watkins, L. W. J. Baillie

**Affiliations:** ^1^ZERO2FIVE Food Industry Centre, Cardiff Metropolitan University, Llandaff Campus, Western Avenue, Cardiff CF5 2YB, UK; ^2^School of Earth and Ocean Sciences, Park Place, Cardiff University, Cardiff CF10 3AT, UK; ^3^Cardiff School of Pharmacy and Pharmaceutical Sciences, King Edward VII Avenue, Cardiff University, Cardiff CF10 3NB, UK

## Abstract

**Background:**

Methane emissions from agriculture are responsible for over 40% of the world's greenhouse gas emissions. In the past, antibiotics were used to control methane production by animals, but concerns over the emergence and spread of antibiotic-resistant bacteria to humans have prompted a search for alternative approaches. Hops are the flowers of the hop plant *Humulus lupulus*. They have been used to feed cattle for many years and are known to contain antibacterial compounds, and their extracts have been shown to kill members of the *Mycobacterium* spp including *Mycobacterium bovis*, the causative agent of bovine tuberculosis as well as a number of human pathogens. In this study, hop extracts were studied for their ability to inhibit methane production from *Methanobrevibacter ruminantium*, a major methane-producing archaeon found in the rumen of cattle.

**Methods:**

*Methanobrevibacter ruminantium* M1^T^ (DSM 1093) was grown at 37°C for 30 days, and the amount of methane produced at different time points during this period was measured using gas chromatography. The archaeon was exposed to commercial hop extracts (tetra-hydro-iso-alpha acid and beta acid) and to aqueous hop extracts of a range of hop variants, and their effect on methane production was determined.

**Results:**

All of the extracts reduced the level of methane production of *M. ruminantium* over the 30-day period compared to the negative control (sterile distilled water). The commercial hop extracts were the most effective at inhibiting methane production over the course of the experiment in contrast to the aqueous extracts, which showed a gradual reduction of inhibition with time.

**Conclusions:**

Hops contain compounds which inhibit methane production. Given that hops can be safely fed to cattle, this raises the possibility of rationally designing a feed strategy which could reduce greenhouse gas emissions and protect against bovine tuberculosis. This study recommends that further research be undertaken to further identifying bioactive components from hops and their efficacy against a range of archaea.

## 1. Introduction

Greenhouse gas emissions (GHG) from ruminants have a significant impact on the global climate. In total, methane comprises an estimated 16% of the total global GHG emissions of which up to 40% are produced by the agricultural sector [1]. Methane is much more detrimental to the environment than carbon dioxide as its warming potential is nearly 25 times greater [2]. Domesticated ruminants, such as cattle, are thought to produce up to 86 million metric tonnes (Tg) of methane per year [3]. Of this total, approximately 55.9 Tg comes from beef cattle, 18.9 Tg from dairy cattle, and 9.5 Tg from sheep and goats [3]. In addition to damaging the environment, the production of methane has a direct effect on the animal depriving it 2-12% of the energy available in the food which it consumes [4].

Ruminant digestion relies on the microflora of the gut; the characterization of which is extremely challenging [5]. We do know that this complex flora includes methanogens, which are archaeon and employ H_2_ to reduce CO_2_ to CH_4_ [6]. While there are a variety of methanogen species, recent analysis has revealed three main groups: *Methanobrevibacter*, which account for 61.6% of rumen archaea (6), *Methanomicrobium*, and the previously uncultured rumen cluster [7, 8].

Controlling ruminant methane production is a complex issue as methanogens are essential for the survival of the ruminant [6]. Previously, antibiotics were used to optimize the ruminant microflora and to improve animal health and productivity, but in 2013, the EU banned the use of antibiotics as animal food supplements due to concerns over the spread of antibiotic resistant bacteria [9]. As a consequence, the agricultural industry is seeking alternative means to control the level of deleterious bacteria in the gut flora of domestic animals.

One approach which is actively being pursued by a number of groups is to screen natural products as in the past they have yielded a number of novel antimicrobials with activity against both animal and human pathogens [10].

One such natural product is *Humulus lupulus*, the flowering plant known generally as hops and which are commonly used in the brewing industry [11]. In addition to imparting the bitter taste which is common of dark beers, the plant also contains a number of antimicrobial compounds such as alpha and beta acids and polyphenolic compounds which are active against Gram-positive spoilage bacteria and thus increase the shelf life of the product [12, 13]. This antibacterial activity against Gram-positive bacteria raises the possibility that hops and their extracts may be able to inhibit the production of methane from methanogenic archaea. A study by Narvaez et al. investigated the effect of supplementing ruminant feed with hops and concluded that hops represented an efficient strategy to reduce methane production and increase energy efficiency for ruminant production [14]. The idea of feeding hops to animals is not new and is commonly used to add value to a brewery waste product [15].

In an earlier study, we observed that hops varieties differed widely in their antimicrobial activity and that processed hops retained detectable amounts of this activity [16]. We thus decided to determine which hop variant was the most effective at inhibiting the ability of *Methanobrevibacter ruminantium* to produce methane.

## 2. Materials

All materials were purchased from Sigma-Aldrich, UK, unless otherwise stated.

Hops were purchased from http://www.themaltmiller.com and had been previously pelleted and stored under vacuum at 3°C.

*Methanobrevibacter ruminantium* M1^T^ (DSM 1093) was purchased from the German Collection of Microorganisms and Cell Cultures DSMZ (Braunschweig, Germany).

Supercritical CO_2_ hop extracts were prepared and supplied by BetaTec Limited, UK.

## 3. Methods

### 3.1. Production of Methanogen Growth Medium

Methanogen growth medium was modified with DSMZ methanobacterium medium, DSMZ media 119; the medium was reduced with FeS rather than cysteine and resazurin which were omitted. Briefly, all products were combined prior to autoclaving apart from bicarbonate, sulfide, sludge fluid, and fatty acid mixture. These were added postautoclaving after the medium had cooled under 80% H_2_ and 20% CO_2_ [17].

The growth medium was dispensed into sterile anaerobic tubes (Bellco Glass Inc., New Jersey, USA) sealed with butyl rubber stoppers and sealed with aluminum crimps. Tubes were stored at 21°C for 7-14 days at which time it was visually inspected for signs of oxidation (color change from black to colorless).

### 3.2. Growth of *M. ruminantium*

*M. ruminantium* was stored in the dark at room temperature within methanogen growth medium. Methane production was measured using gas chromatography as described below.

### 3.3. Aqueous Hop Extraction

Upon delivery, hop material was stored at 3°C within an airtight container. Prior to extraction, 5 g of pellets was mechanically macerated and suspended, with mixing in 200 ml of sterile deionized water and incubated at 100°C for 60 mins. The hop solutions were allowed to cool prior to coarse filtration using a 1 mm gauze. The resulting extract was purged with 100% N_2_ and stored in anoxic tubes prior to testing.

### 3.4. Test Conditions

Following extraction, 1 ml of unsterilized hop extract (aqueous or CO_2_) was added to 15 ml methanogen growth media 72 hours after inoculation with *M. ruminantium*. Controls consisted of 20 ml growth media with 1 ml of sterile deionized water or 1 ml of 3 mg/ml metronidazole, which was added at day 0.

Methane production was used as a measure of growth, and gas chromatography was employed. Samples were taken from each test and control tube during a 6-hour period on day 10, 20, and 30.

### 3.5. Measurement of Methane Production Using Gas Chromatography

Headspace gases were analyzed using a modified PerkinElmer/Arnel Clarus 500 natural gas analyser (PerkinElmer, Waltham, MA, USA) equipped with a flame ionization detector (FID) (16).

The oven temperature was 110°C with the FID at 250°C, and the carrier gas was helium.

### 3.6. Calibration of Gas Chromatogram

Three standard mixed gases (Scott Specialty Gases, Plumsteadville, Pennsylvania, USA) were used to calibrate the system.

## 4. Results

Due to the fastidious growth requirements of *M. ruminantium*, we were only able to culture the methanogen in liquid medium. The heavy pigmentation of this medium meant that we were unable to monitor the progress of growth using common spectroscopic methods and thus were forced to employ the methane production as a measure of growth [17].

**Figure 1:**
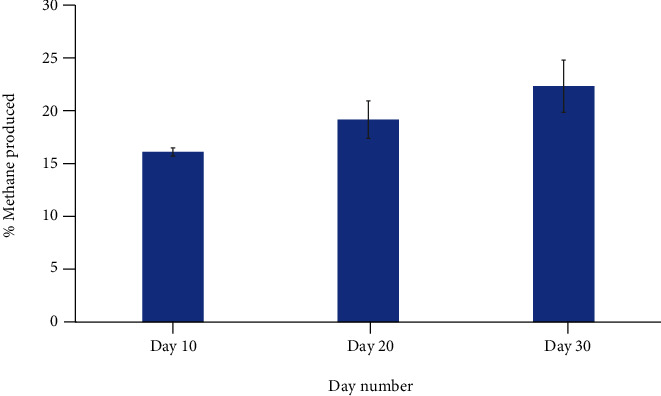
The percentage methane produced by *Methanobrevibacter ruminantium* M1^T^ (DSM 1093). Gas samples were taken at 10-, 20-, and 30-day time points and each data point is an average of 3 separate experiments ± SE.

To characterize the time course and magnitude of methane production from *M. ruminantium*, we cultured the methanogen over a 30-day period and measured the level of methane production at 10-day intervals. As can be seen in Figure 1, the level of methane increased with time.

This methanogen is known to be susceptible to the antibiotic metronidazole, so we next determined the ability of different concentrations of the antibiotic to inhibit methane production. Metronidazole was added at day 0 and incubated with the bacterium for 30 days. At which time, the concentration of methane was measured and compared to an untreated control.

**Figure 2:**
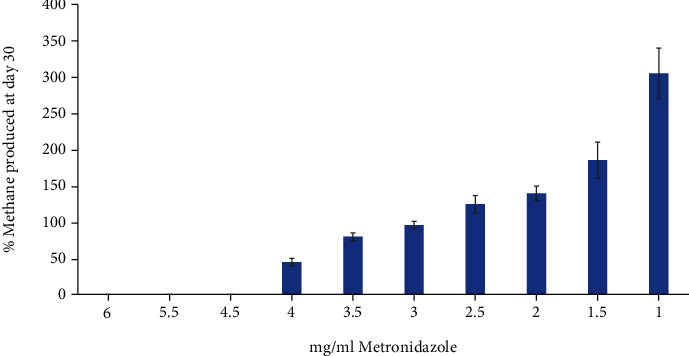
The effect of metronidazole on the methane production of *M. ruminantium* following 30 days of incubation. Metronidazole at varying concentrations was added to cultures of *M. ruminantium* and incubated at 37°C for 30 days. On day 30, the methane concentration was measured and compared to an untreated control, which at day 30 gave an average concentration of 22.7% methane (Figure 1). Results are an average of 3 separate repeats ± SE.

As can be seen from Figure 2, the ability of metronidazole to inhibit methane production was concentration dependent, with 4.5 mg/ml of antibiotic being sufficient to inhibit detectable methane production.

We next compared the ability of five different hop variants and two commercially available hop extracts to inhibit the methane production of *M. ruminantium*. Metronidazole at a concentration of 4.5 mg/ml was included as a control as was a culture treated with 1 ml of sterile deionized water. As can be seen from Table 1, all of the hop extracts inhibited the production of methane to varying degrees across all of the time points.

The results observed in Table 1 indicate that at day 10, the greatest level of inhibition was observed in the aqueous hop extract samples. The Willamette variant presented the highest level of inhibition. Statistical analysis using the Student's *t*-test showed that there was no correlation (*p* > 0.05) between the stated alpha and beta acid concentration of the hop variants and the percentage reduction in methane production. Interestingly, the methane production increased over time, and at day 30, the magnum hop variant had only a 38% reduction in methane production. This increase in methane production suggests that the inhibitory effect is transient and reflects either a reduction in the concentration of the inhibitory element with time, an increase in the resistance of the methanogens to the active ingredients or inhibition of growth leading reduced substrate use over a longer period of time.

In comparison, the commercial hop extracts achieved a consistently high reduction in methane production with the beta acid extract (10% *w*/*w*) being the most efficient. The effect of the commercial extracts increased with time, and by day 30, the level of methane reduction was comparable to the metronidazole control.

## 5. Discussion

In this study, we have shown that hop variants differed in their ability to inhibit methane production of *Methanobrevibacter ruminantium.* Of the variants examined, Willamette was initially the most active but this activity reduced with time. This increase in methane production suggests that the inhibitory effect reflects either a reduction or inactivation in the concentration of the inhibitory element with time or an increase in the resistance of the bacteria to the active ingredients.

Research by several groups has identified several organisms, such as *Lactobacillus brevis*, *Lactobacillus lindneri* [18], and *Pediococcus damnosus* that can develop resistance to hop acids through multiple mechanisms [19, 20]. Hops primarily exert a bacteriostatic effect with the hop acids (lupulone and humulone) causing inhibition of transport mechanisms across the cell membrane [21]. In *Lactobacillus brevis*, it was reported that trans-isohumulone acts as an ionophore; catalyzing the electroneutral influx of undissociated isohumulone, internal dissociation of (H^+^)-isohumulone, and efflux of the complex of isohumulone reduces the uptake of leucine [20, 22, 23].

The ability of the commercial beta acid preparation to inhibit the majority of methane production across the time course of the study suggests that 10% represents an effective inhibitory concentration. The observation that the 95% alpha acid preparation was less effective than the 10% beta acid at 10 days indicates that the beta acid preparation is more effective at inhibiting methane production from this archaeon.

It is interesting that Willamette, the most inhibitory of the hop variants tested, had one of the lowest hop acid contents, which suggests that other factors may have contributed to the ability of this variety to inhibit methane production. Indeed, it may also be the case that other compounds found within hops are synergistically antimicrobial with the alpha and beta acids. Previous studies have identified the polyphenolic content of hops such as xanthohumol and its related prenylflavonoids as having antimicrobial activity against a wide range of pathogens [24]. For the hop variants tested in this study, the polyphenolic content was not measured. Thus, it may be the case that the polyphenolic content of the hop variants tested may have either a direct of synergistic antimicrobial effect with the alpha and beta acids. Identifying all of the antimicrobial compounds in hops and their synergistic activity is an area which requires more research as highlighted in our previous study [16].

We previously found that by replicating the brewing process, hops still retained a significant amount of antimicrobial activity (unpublished data). Thus, it may be the case that waste hops discarded following the brewing process may represent a valuable resource not just as a renewable animal feed but also as a natural food supplement to reduce methane emissions. Indeed, this conclusion has already been considered by other groups for several industrial by-products including those of the brewing industry [25–27]. More research would need to be undertaken to identify the most efficacious hop variant and the variant which retains the highest amount of antimicrobial activity following the brewing process.

## 6. Conclusions

We can thus conclude that hop extracts contain compounds which inhibit the production of methane produced by *Methanobrevibacter ruminantium* and the level of these compounds varies between varieties. Thus, more research needs to be undertaken to identify the specific antimicrobial compounds, their individual and synergistic activity, and the differences between hop variants.

Based on these results, the interaction of hop extracts with *M. ruminantium* and other methanogens within the rumen would highlight the efficacy of hop variants and their ability to be used as a method of methane reduction.

Hop extracts have the potential to be developed as a food supplement for cattle with a view to improving energy extraction and reducing the production of environmentally damaging greenhouse gases.

## Figures and Tables

**Table 1:** The percentage reduction of methane by aqueous and commercial hop extracts, metronidazole (4.5 mg/ml), and water compared to an untreated control at 10-day time points over 30 days. Results are an average of 3 separate repeats.

Controls	Percentage methane produced at		
	Day 10	Day 20	Day 30
Metronidazole 4.5 mg/ml	0%	0%	0%
Sterile distilled water	100%	100%	100%
Hop extracts	Percentage methane produced		
Citra	3%	32%	42%
Fuggles	2%	26%	49%
Willamette	2%	20%	50%
Magnum	6%	39%	62%
Northdown	5%	23%	68%
Commercial hop extracts	Percentage methane produced		
Tetra-hydro-iso-alpha acid	29%	1%	1%
Beta acid	9%	1%	1%

## Data Availability

Data is available for all figures and tables used.
